# Deep learning computer-aided detection system for pneumonia in febrile neutropenia patients: a diagnostic cohort study

**DOI:** 10.1186/s12890-021-01768-0

**Published:** 2021-12-07

**Authors:** Eui Jin Hwang, Jong Hyuk Lee, Jae Hyun Kim, Woo Hyeon Lim, Jin Mo Goo, Chang Min Park

**Affiliations:** 1 Department of Radiology, Seoul National University College of Medicine, 101 Daehak-ro, Jongno-gu, Seoul, 03080 Korea; 2 Namwon Medical Center, 365 Chungjeong-ro, Namwon, 55726 Jeollabuk-do Korea; 3 Institute of Medical and Biological Engineering, Medical Research Center, Seoul National University, 103 Daehak-ro, Jongno-gu, Seoul, 03080 Korea

**Keywords:** Radiography, Thoracic, Deep learning, Artificial intelligence, Pneumonia, Febrile neutropenia

## Abstract

**Background:**

Diagnosis of pneumonia is critical in managing patients with febrile neutropenia (FN), however, chest X-ray (CXR) has limited performance in the detection of pneumonia. We aimed to evaluate the performance of a deep learning-based computer-aided detection (CAD) system in pneumonia detection in the CXRs of consecutive FN patients and investigated whether CAD could improve radiologists’ diagnostic performance when used as a second reader.

**Methods:**

CXRs of patients with FN (a body temperature ≥ 38.3 °C, or a sustained body temperature ≥ 38.0 °C for an hour; absolute neutrophil count < 500/mm^3^) obtained between January and December 2017 were consecutively included, from a single tertiary referral hospital. Reference standards for the diagnosis of pneumonia were defined by consensus of two thoracic radiologists after reviewing medical records and CXRs. A commercialized, deep learning-based CAD system was retrospectively applied to detect pulmonary infiltrates on CXRs. For comparing performance, five radiologists independently interpreted CXRs initially without the CAD results (radiologist-alone interpretation), followed by the interpretation with CAD. The sensitivities and specificities for detection of pneumonia were compared between radiologist-alone interpretation and interpretation with CAD. The standalone performance of the CAD was also evaluated, using area under the receiver operating characteristic curve (AUC), sensitivity, and specificity. Moreover, sensitivity and specificity of standalone CAD were compared with those of radiologist-alone interpretation.

**Results:**

Among 525 CXRs from 413 patients (52.3% men; median age 59 years), pneumonia was diagnosed in 128 (24.4%) CXRs. In the interpretation with CAD, average sensitivity of radiologists was significantly improved (75.4% to 79.4%, *P* = 0.003) while their specificity remained similar (75.4% to 76.8%, *P* = 0.101), compared to radiologist-alone interpretation. The CAD exhibited AUC, sensitivity, and specificity of 0.895, 88.3%, and 68.3%, respectively. The standalone CAD exhibited higher sensitivity (86.6% vs. 75.2%, *P* < 0.001) and lower specificity (64.8% vs. 75.4%, *P* < 0.001) compared to radiologist-alone interpretation.

**Conclusions:**

In patients with FN, the deep learning-based CAD system exhibited radiologist-level performance in detecting pneumonia on CXRs and enhanced radiologists’ performance.

**Supplementary Information:**

The online version contains supplementary material available at 10.1186/s12890-021-01768-0.

## Background

Febrile neutropenia (FN) is observed in approximately 1% of patients receiving chemotherapy and has a mortality rate of approximately 10% [1–5]. Therefore, it is considered as a medical emergency that requires timely diagnosis and management [1, 5]. Investigation of the infection focus is the key component of diagnostic work-ups in these patients, and pneumonia is one of the most common and important causes of FN [6–9]. Indeed, pneumonia is associated with a higher rate of treatment failure, longer hospitalization, and increased mortality in FN patients [1–3, 10].

Chest X-ray (CXR) is the initial radiological examination for the evaluation of pneumonia at low cost and wide availability and thus considered a routine investigation in FN patients [1, 11–13]. However, a poor immune response may delay the development of radiographic infiltrates, causing difficulty in the sensitive detection of early pneumonia on CXR [14–16]. Furthermore, CXRs have high inter-observer variability among less-experienced readers [17, 18], and timely interpretation of CXRs by expert radiologists is not always possible. In this regard, a computer-aided detection (CAD) system that can identify pulmonary infiltrates suggestive of pneumonia on CXRs may help managing FN patients.

Recently, deep learning technologies showed promising performance regarding the detection of pneumonia on CXRs [19–22]. However, those studies tested the performance of CAD using conveniently collected datasets, with enriched prevalence of pneumonia [19–22] and limited radiologic diversity (i.e., composed of CXRs with pneumonia and clearly normal CXRs) [19–21], which is definitely far from the situation in the real-world setting. Thus, it remains difficult to believe that deep learning-based CAD truly proved their clinical usefulness in pneumonia detection [22]. In this context, external validation in consecutive patients in a real clinical setting is necessary to confirm the real-world accuracy and clinical usefulness of deep learning-based CAD.

Therefore, we evaluated the performance of deep learning-based CAD in pneumonia detection in the CXRs of consecutive FN patients and investigated whether CAD could improve radiologists’ diagnostic performance when used as a second reader.

## Methods

This single-center, retrospective study was approved by the institutional review board. The requirement for patients’ informed consents was waived.

### Study population

We retrospectively included patients from a single tertiary referral hospital if they met the following inclusion criteria: (a) patients with a body temperature ≥ 38.3 °C or a sustained body temperature ≥ 38.0 °C for an hour between January and December 2017 [13]; (b) patients with absolute neutrophil count (ANC) < 500/mm^3^ in peripheral blood on the day of fever [13]; and (c) patients who underwent CXRs on the day of fever.

In cases of multiple CXRs from a single patient, only the first CXR was included to represent the episode of FN. However, if there was an afebrile period of 30 days or longer between the days of fever, they were regarded as different episodes of FN and the first CXR in each episode was included.

### CXR acquisition and CAD system

All CXRs were obtained using a fixed radiography system (Digital Diagnost, Philips Healthcare, Eindhoven, The Netherlands) or a portable radiography scanner (DRX-revolution, Carestream Health, Rochester, NY), depending on patients’ condition. CXRs from a fixed radiography system were obtained in the erect position with posteroanterior projection, while CXRs from a portable radiography scanner were obtained in the supine or sitting position with anteroposterior projection.

We used a commercialized, regulatory-approved deep learning-based CAD (Lunit INSIGHT CXR 2, version 2.0.0.0, Lunit Inc., Seoul, Korea) to evaluate CXRs. The CAD was designed to detect radiographic abnormalities, including pulmonary nodules or masses, pulmonary infiltrates, and pneumothorax on a single frontal CXR. The CAD was initially trained using 54,221 normal CXRs and 35,613 abnormal CXRs including 6,903 CXRs from patients with pneumonia (see supplementary material for further information) [21].

**Fig. 1:**
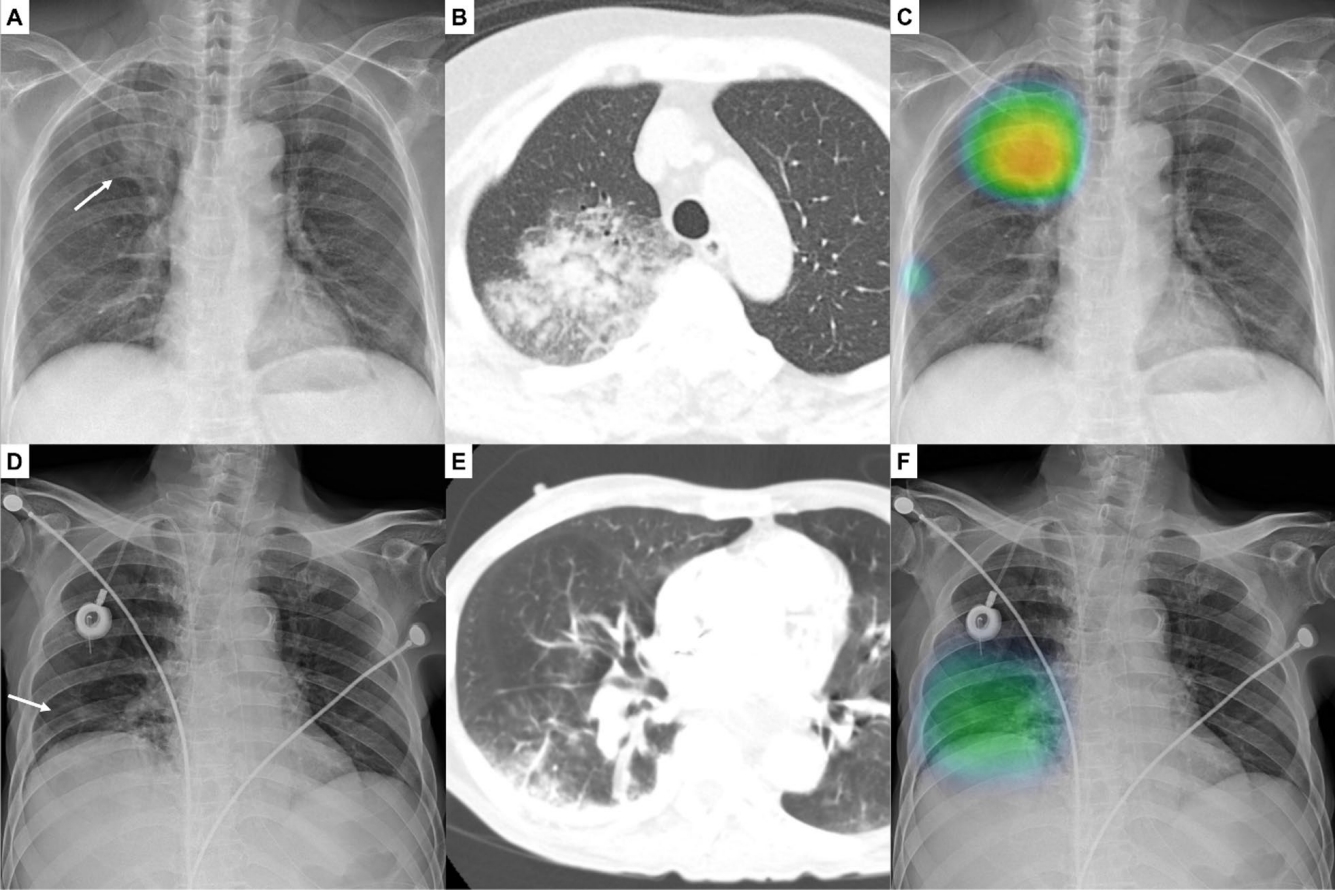
Identification of pneumonia on chest X-ray (CXR) using the computer-aided detection (CAD) system

The CAD provided probability scores for the presence of each target abnormality between 0 and 100% for a CXR. When the probability score was 15% or greater, the CAD also provided a heat map overlaid on the CXR for the localization of the abnormality (Fig. 1). In the present study, we used only probability scores for pulmonary infiltrate (see supplementary material for further information).

A CXR of a 68-year-old woman with febrile neutropenia (absolute neutrophil count, 366/mm^3^; body temperature, 39.1 °C) showing increased opacity in the right upper lung (A, arrow). A chest computed tomographic scan obtained on the same day showing the corresponding consolidation and ground-glass opacity lesions in the upper lobe of the right lung, suggestive of pneumonia (B). The CAD system correctly identified the abnormality with a probability score of 84% (C). In the reader test, three of five radiologists correctly identified the pulmonary infiltrate on the CXR in the radiologist-alone interpretation, and all five radiologists identified the lesion in the interpretation with CAD.

A CXR of a 69-year-old man with febrile neutropenia (absolute neutrophil count, 489/mm^3^; body temperature, 38.9 °C) showing subtle increased opacity in the right lower lung (D). A chest computed tomographic scan showing the corresponding consolidation and ground-glass opacity lesions in the lower lobe of the right lung, suggestive of pneumonia (E). The CAD system correctly identified the abnormality with a probability score of 51% (F). In the reader test, two of five radiologists correctly identified the pulmonary infiltrate on the CXR in the radiologist-alone interpretation, and four radiologists including two radiologists who initially could not recognize the lesion correctly identified the lesion in the interpretation with CAD.

### Reference standard

Two thoracic radiologists (C.M.P. and E.J.H., 21 and 9 years of experience in interpreting CXR and chest CT) who were blinded to the CAD results reviewed patients’ medical records and radiological, microbial, and laboratory examination results to define, in consensus, the clinical diagnosis of pneumonia at the time of CXR. The presence of clinical features suggestive of respiratory infection with demonstrable pulmonary infiltrate by CXR or CT was regarded as a clinical diagnosis of pneumonia, regardless of the microbiological identification of the causative pathogen [23]. When a pulmonary infiltrate was identified on CT but invisible on CXR, such case was defined as positive for pneumonia based on CT findings.

### Reader test

To compare the performance of CAD with that of radiologists and to evaluate whether CAD can enhance the performance of radiologists, we conducted a retrospective reader test. Five board-certified radiologists (general radiologists without subspecialty training for thoracic radiology, 1–3 years of experience after finishing residency) participated in the reader test, and 50% of CXRs among the entire cohort were randomly sampled for the test.

Each radiologist independently interpreted CXRs to classify them into those with or without pulmonary infiltrates suggestive of pneumonia. First, radiologists read the CXRs without CAD results (radiologist-alone interpretation). After finishing the first reading session for all CXRs, they re-interpreted the CXRs with the corresponding CAD results and were allowed to change their initial decision as needed (interpretation with CAD). The radiologists were informed that all CXRs were obtained from FN patients; however, they were blinded to other clinical or laboratory information.

### Subgroup analyses

For a more solid reference standard for the presence of pulmonary infiltrates, we separately evaluated the performance of CAD in patients with available chest CT obtained within 3 days from the CXRs with reference to CTs for the presence of pulmonary infiltrates.

To investigate the performance of CAD in patients with different clinical characteristics, we evaluated the performance of CAD in the following subgroup populations: (a) male vs. female patients; (b) patients aged < 60 years vs. ≥ 60 years; (c) CXRs from a fixed radiography system vs. CXRs from a portable radiography scanner.

### Statistical analyses

The discriminative performance of CAD (ability to separate CXRs with and without pneumonia) was evaluated using the area under the receiver operating characteristic curve (AUC). The sensitivity and specificity of CAD were determined at the pre-defined threshold (probability score of 15%) recommended by the manufacturer. A comparison of AUCs of CAD between subgroups was performed according to the method by DeLong [24], while the chi-square test was used to compare the sensitivities and specificities of CAD between subgroups.

To compare the performance between radiologists and CAD, the sensitivity and specificity of CAD at the pre-defined threshold were compared with those of individual radiologists using generalized estimating equations, to consider the clustering effects caused by multiple CXRs obtained from a single patient and multiple interpretations by radiologists for a single CXR [25]. The sensitivities and specificities of radiologists in radiologist-alone interpretation and interpretation with CAD were also compared to investigate whether or not CAD can enhance radiologists’ detection performance.

Calibration (degree of agreement between the predicted probabilities by CAD versus the observed probability of pneumonia) of CAD was evaluated by constructing a calibration plot. Inter-reader agreement among radiologists was evaluated using the Fleiss’ kappa coefficient.

All statistical analyses were performed using R (version 3.6.3, R project for statistical computing, Vienna, Austria). A *P *value of less than 0.05 was considered to indicate a statistically significant difference.

## Results

### Demographic and clinical information

A total of 525 CXRs obtained from 413 patients (216 men and 197 women; median age, 59 years [interquartile range (IQR), 48–67]) were analyzed in the present study. Based on the information in medical records, pneumonia was diagnosed in 128 (24.4%) FN episodes. Detailed demographic and clinical information are described in Table 1.

**Table 1:** Demographic and clinical information

Variable	Data
Male patient (%)^a^	52.3% (216/413)
Age (year)^b^	59 (48–67)
Number of CXR per patient^a^	
1 CXR per patient	79.9% (330/413)
2 CXRs per patient	13.6% (56/413)
3 CXRs per patient	6.1% (25/413)
4 CXRs per patient	0.5% (2/413)
Underlying disease of medical history^a^	
Hematologic malignancy or lymphoma	60.8% (251/413)
Solid organ malignancy	26.4% (109/413)
Hematopoietic stem cell transplantation	17.4% (72/413)
Solid organ transplantation	1.7% (7/413)
ANC (count per mm^3^)^b^	36 (0–166)
Peak body temperature (°C)^b^	38.9 (38.5–39.3)
Site of infection^c^	
Pneumonia	24.4% (128/525)
Gastrointestinal tract	6.9% (36/525)
Bone or soft tissue	4.2% (22/525)
Oral cavity	1.9% (10/525)
Bloodstream infection	1.7% (9/525)
Urinary tract	1.3% (7/525)
Biliary tree	0.8% (4/525)
Unidentifiable infection focus	62.1% (326/525)
CXRs from fixed radiography unit	27.8% (146/525)
CXRs from portable radiography scanner	72.2% (379/525)

CAD, computer-aided detection; CXR, chest X-ray; ANC, absolute neutrophil count

^a^Data indicate proportions among entire patients (numerators/denominators)

^b^Data indicate median (interquartile ranges) values

^c^Data indicate proportions among entire episodes of febrile neutropenia (numerators/denominators)

### Standalone performance of the CAD

**Fig. 2:**
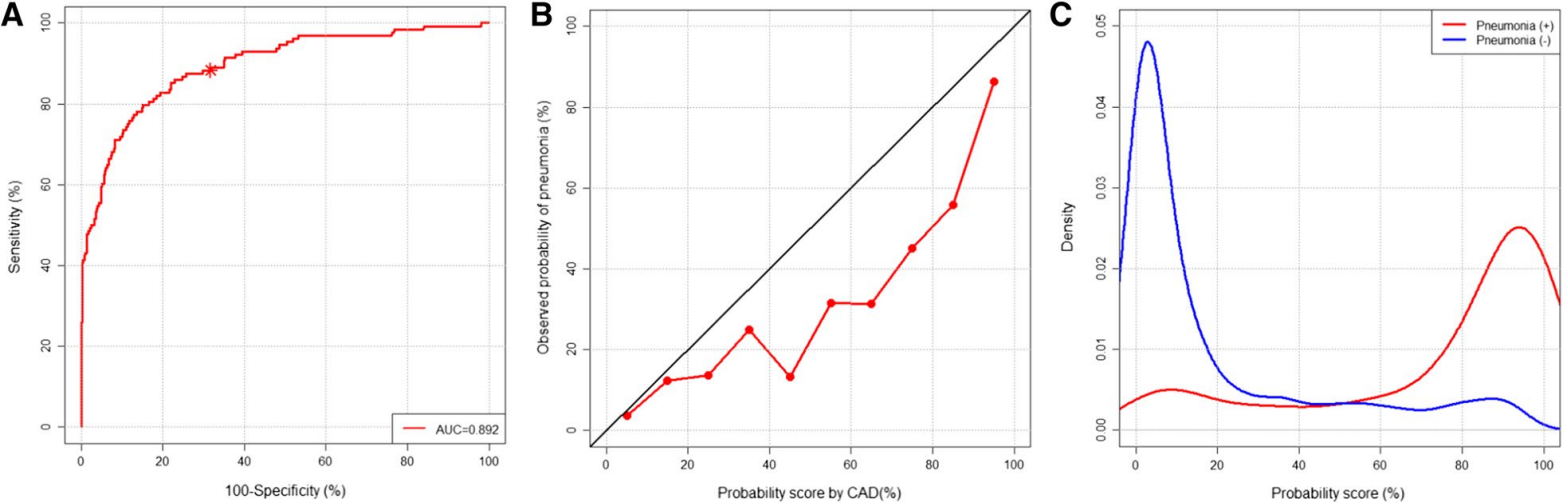
Performance of the computer-aided detection (CAD) system in all patients

The performance of CAD is summarized in Figure three. The CAD exhibited an AUC of 0.892 (95% confidence interval [CI], 0.851–0.926) for the identification of pneumonia on CXRs. At the pre-defined threshold, CAD exhibited a sensitivity and specificity of 88.3% (95% CI, 82.7–93.9%) and 68.3% (95% CI, 63.7–72.8%), respectively (Fig. 2).

Regarding calibration, CAD tended to overestimate the probability of pneumonia (Fig. 2). Data of the sensitivities and specificities of CAD at different thresholds are listed in Additional file 1: Table S1 in the supplementary material.

For all patients, the CAD system showed an area under the receiver operating characteristic curve of 0.892 for the identification of pneumonia in the chest X-ray. At a pre-defined threshold probability score of 15%, the sensitivity and specificity of the CAD system were 88.3% and 68.3%, respectively (A). The calibration plot (B) shows that the CAD system tended to overestimate the probability of pneumonia. The density plot of the probability score from the CAD system according to the presence of pneumonia (C) shows that the CAD system can relatively clearly separate CXRs with pneumonia from those without pneumonia.

### Reader test

**Fig. 3:**
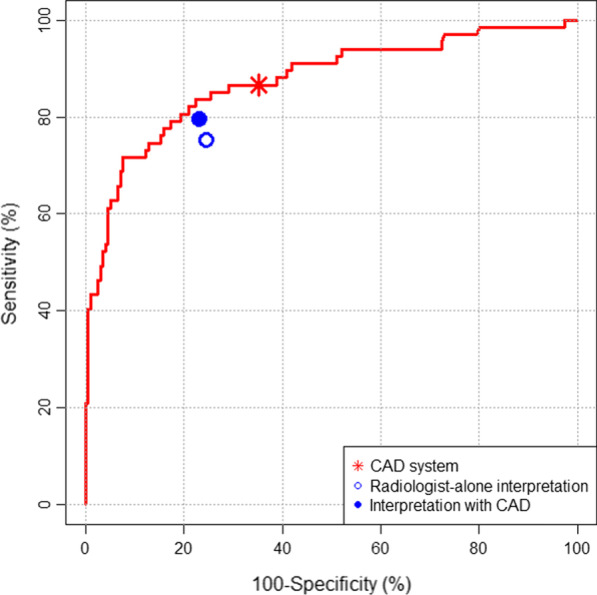
Performance of the computer-aided detection (CAD) system and radiologists in the reader test

A total of 263 (50.1%) CXRs were randomly sampled for the reader test. Among them, 67 (25.5%) were obtained from patients with pneumonia. Data of the performances of CAD and each radiologist are listed in Table 2 and Fig. 3.

**Table 2:** Performances of the computer-aided detection system and radiologists in the reader test

Reader	Sensitivity	*P *value	Specificity	*P *value
CAD system	86.6% (58/67, 76.2–92.9%)^a^	Reference	64.8% (127/196, 58.1–71.5%)^a^	Reference
Radiologist-alone interpretation				
Average	75.2% (252/335, 65.7–82.8%)	< 0.001^b^	75.4% (739/980, 70.9–79.4%)	< 0.001^b^
Radiologist A (8 years experience)	79.1% (53/67, 67.7–87.2%)	0.019^b^	76.0% (149/196, 69.5–81.5%)	< 0.001^b^
Radiologist B (6 years experience)	71.6% (48/67, 59.8–81.1%)	0.001^b^	81.1% (159/196, 75.0–86.0%)	< 0.001^b^
Radiologist C (7 years experience)	64.2% (43/67, 52.1–74.7%)	< 0.001^b^	88.3% (173/196, 83.0–92.1%)	< 0.001^b^
Radiologist D (7 years experience)	86.6% (58/67, 79.2–92.9%)	> 0.999^b^	44.9% (88/196, 38.1–51.9%)	< 0.001^b^
Radiologist E (5 years experience)	74.6% (50/67, 62.3–83.6%)	0.003^b^	86.7% (170/196, 81.2–90.8%)	< 0.001^b^
Interpretation with CAD				
Average	79.4% (266/335, 69.9–86.5%)	0.003^c^	76.8% (753/980, 72.1–81.0%)	0.101^c^
Radiologist A (8 years experience)	85.1% (57/67, 74.4–91.8%)	0.039^c^	75.5 (148/196, 69.0–81.0%)	0.841^c^
Radiologist B (6 years experience)	76.1% (51/67, 64.5–84.8%)	0.174^c^	79.6% (156/196, 73.4–84.7%)	0.365^c^
Radiologist C (7 years experience)	73.1% (49/67, 61.3–82.4%)	0.010^c^	87.8% (172/196, 82.4–91.7%)	0.763^c^
Radiologist D (7 years experience)	86.6% (58/67, 79.2–92.9%)	> 0.999^c^	53.6% (105/196, 46.6–60.4%)	< 0.001^c^
Radiologist E (5 years experience)	76.1% (51/67, 64.5–84.8%)	0.314^c^	87.8% (172/196, 82.4–91.7%)	0.316^c^

Numbers in parentheses indicate numerators/denominators, 95% confidence intervals

The experience of each radiologist indicates the length of their experiences in the interpretation of chest X-rays

CAD, computer-aided detection

^a^Performance of the CAD system at the predefined threshold (probability score of 15%)

^b^Comparison of performance between the CAD system and the radiologist-alone interpretation

^c^Comparison of performance between radiologist-alone interpretation and interpretation with CAD

In the radiologist-alone interpretation, radiologists exhibited average sensitivity and specificity of 75.2% and 75.4%, respectively. At the pre-defined threshold, the CAD exhibited significantly higher sensitivity (86.6% vs. 75.2%, *P* < 0.001) and lower specificity (64.8% vs. 75.4%, *P* < 0.001) than radiologist-alone interpretation. Regarding individual radiologists, the CAD showed significantly higher sensitivity and specificity than four and one radiologist, respectively. Meanwhile, the CAD showed significantly lower specificity than four radiologists (Table 2).

In the interpretation with CAD, average sensitivity of radiologists was significantly improved (75.4% to 79.4%, *P* = 0.003) while their specificity remained similar (75.4% to 76.8%, *P* = 0.101), compared to radiologist-alone interpretation. Regarding individual radiologists, significant improvements in sensitivity and specificity were observed in two and one radiologist, respectively (Table 2).

Analyses after exclusion of radiologists who showed outlier sensitivity and specificity exhibited similar results with those including all radiologists (Additional file 1: Table S2 in the supplementary material).

Regarding the inter-reader agreement among radiologists, interpretation with CAD exhibited better agreement (Fleiss’ kappa coefficient, 0.638 [95% CI, 0.600–0676]) than the radiologist-alone interpretation (Fleiss’ kappa coefficient, 0.546 [95% CI, 0.507–0.584]).

In the reader test, the CAD system showed an area under the receiver operating characteristic curve of 0.895 for the identification of pneumonia in the chest X-ray. At a pre-defined threshold probability score of 15%, the sensitivity and specificity of the CAD system were 86.6% and 64.8%, respectively. In the radiologist-alone interpretation, radiologists exhibited average sensitivity and specificity of 75.2% and 75.4%, respectively. In the interpretation with CAD, average sensitivity of radiologists was significantly improved (79.4%), at the similar specificity (76.8%).

### Subgroup analyses

**Fig. 4:**
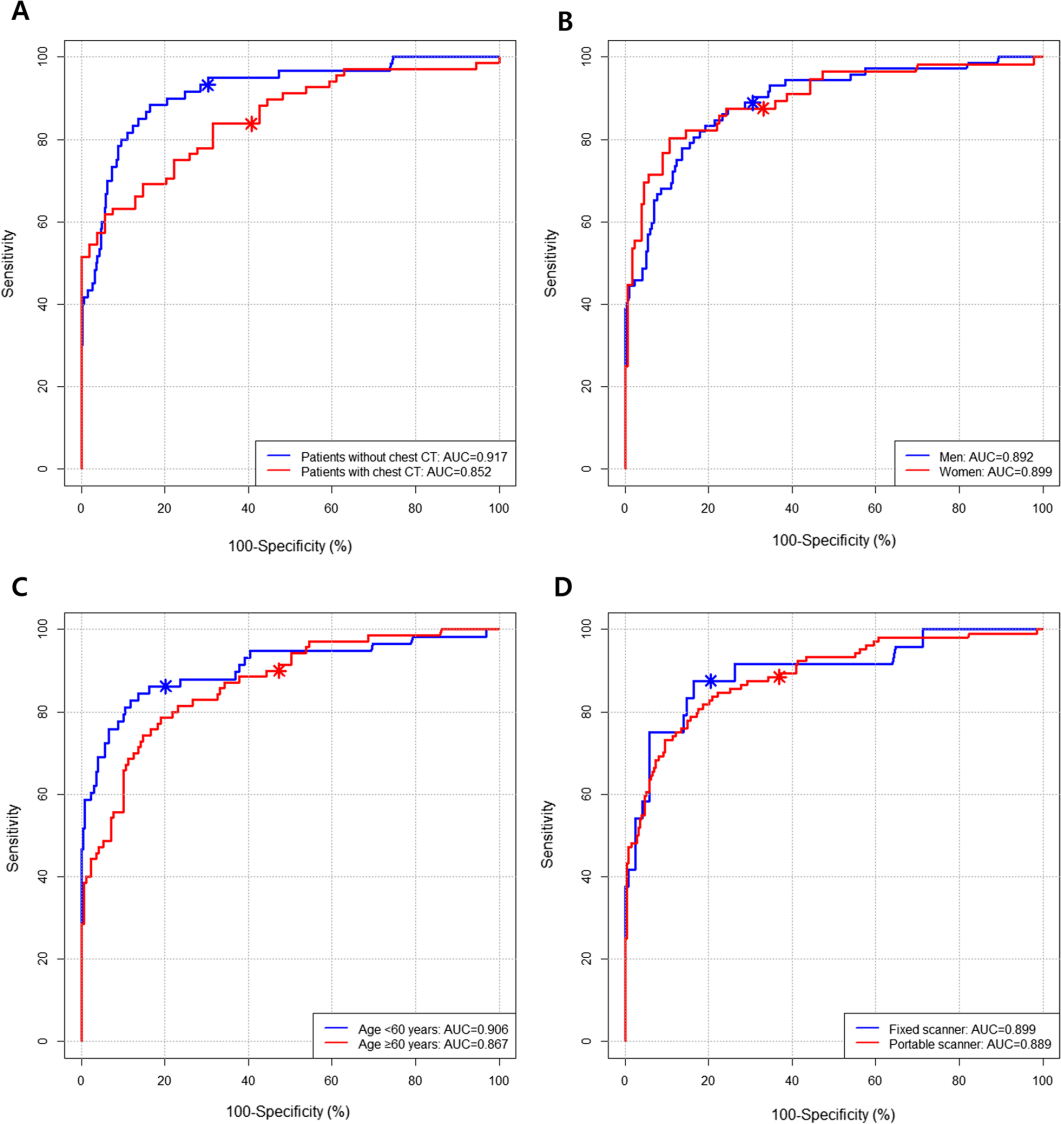
Performance of the computer-aided detection (CAD) system in different subgroups

The discriminative performance of CAD in different subgroups is shown in Table 3 and Fig. 4.

**Table 3:** Performance of the computer-aided detection system in different subgroups

Patient groups	AUC	Sensitivity	Specificity
All patients (n = 525)	0.892 (0.851–0.926)	88.3% (113/128, 82.7–93.9%)	68.3% (271/397, 63.7%–72.8%)
Patients with chest CT scan (n = 122)	0.852 (0.776–0.909)	83.8% (57/68, 75.1–92.6%)	59.3% (32/54, 46.2–72.4%)
Patients without chest CT scan (n = 403)	0.917 (0.886–0.942)	93.3% (56/60, 87.0–99.6%)	69.7% (239/343, 64.8–74.5%)
*P *value^a^	0.099	0.095	0.126
Men (n = 291)	0.892 (0.851–0.926)	88.9% (64/72, 81.6–96.1%)	69.4% (152/219, 63.3–75.5%)
Women (n = 234)	0.899 (0.853–0.934)	87.5% (49/56, 78.8–96.2%)	66.9% (119/178, 59.9–73.8%)
*P *value^b^	0.854	0.809	0.587
Age < 60 years (n = 286)	0.906 (0.866–0.937)	86.2% (50/58, 77.3–95.1%)	79.8% (182/228, 74.6–85.0%)
Age ≥ 60 years (n = 239)	0.867 (0.818–0.908)	90.0% (63/70, 83.0–97.0%)	52.7% (89/169, 45.1–60.2%)
*P *value^c^	0.300	0.507	< 0.001
CXRs from a fixed scanner (n = 146)	0.899 (0.838–0.943)	87.5% (21/24, 74.3–100%)	79.5% (97/122, 72.3–86.7%)
CXRs from a portable scanner (n = 379)	0.889 (0.853–0.919)	88.5% (92/104, 82.3–94.6%)	63.3% (174/275, 57.3–68.9%)
*P *value^d^	0.831	0.895	0.001

Numbers in parentheses indicate numerators/denominators, 95% confidence intervals

AUC, area under the receiver operating characteristic curve; CT, computed tomography; CXR, chest X-ray

^a^Comparison of performance between patients with and without a chest CT scan

^b^Comparison of performance between men and women

^c^Comparison of performance between patients aged < 60 years and those aged ≥ 60 years

^d^Comparison of performance between CXRs from a fixed scanner and CXRs from a portable scanner

Chest CTs obtained within 3 days from the CXRs were available in 122 (23.2%) CXRs. Based on CT findings, pulmonary infiltrates suggestive of pneumonia were identified in 68 of 122 cases (55.7%). Among the pulmonary infiltrates, 57 were retrospectively visible on CXRs, while the other 11 were invisible on CXRs, even after correlation with chest CT findings. With the reference standard of chest CT, the CAD system exhibited an AUC of 0.852 (95% CI, 0.776–0.909). At the pre-defined threshold, CAD exhibited sensitivity and specificity of 83.8% (95% CI, 75.1–92.6%) and 59.3% (95% CI, 46.2–72.4%), respectively. When compared with patients without chest CT (AUC, 0.917 [*P* = 0.099]; sensitivity, 93.3% [*P* = 0.095]; specificity, 69.7% [*P* = 0.126]), CAD showed a slightly lower performance in patients with chest CT, without any significant difference.

The CAD showed similar AUCs between men vs. women (0.892 vs. 0.899; *P* = 0.854), patients aged < 60 years vs. ≥ 60 years (0.906 vs. 0.867; *P* = 0.300), and CXRs from a fixed radiography system vs. CXRs from a portable radiography scanner (0.899 vs. 0.899; *P* = 0.831). However, the specificity of CAD was significantly higher in patients aged < 60 years than in those aged ≥ 60 years (79.8% vs. 52.7%; *P* < 0.001) and in CXRs from the fixed radiography system than in CXRs from the portable radiography scanner (79.5% vs. 63.1%; *P* = 0.001).

The CAD system exhibited a slightly lower area under the receiver operating characteristic curve (AUC: 0.852 vs. 0.917; *P* = 0.10), sensitivity (83.8% vs. 93.3%, *P* = 0.10), and specificity (59.3% vs. 69.7%, *P* = 0.13) in patients with a chest CT scan than in those without a chest CT scan, without a significant difference (A). The CAD system showed similar AUCs between men and women (0.892 vs. 0.899; *P* = 0.854; B), patients aged < 60 years and ≥ 60 years (0.906 vs. 0.867; *P* = 0.300; C), and chest X-rays (CXRs) from a fixed radiography system and CXRs from a portable radiography scanner (0.899 vs. 0.899; *P* = 0.83; D). However, the specificity of the CAD system was significantly higher in patients aged < 60 years than in those aged ≥ 60 years (79.8% vs. 52.7%; *P* < 0.001; C), and CXRs from a fixed radiography system than CXRs from a portable radiography scanner (79.5% vs. 63.1%; *P* = 0.001; D).

## Discussion

In our study, the deep learning-based CAD system showed good performance (sensitivity, 88.3%; specificity, 68.3%) in the identification of CXRs with pneumonia in consecutive febrile neutropenia patients. When compared with radiologists, the CAD system exhibited significantly higher sensitivity (86.6% vs. 75.2%) but lower specificity (64.8% vs. 75.4%). In the interpretation assisted by the CAD system, radiologists exhibited improvement in sensitivity (75.2% to 79.4%) at similar specificity (75.4% to 76.8%).

Symptoms suggestive of respiratory infection and pulmonary infiltrates on CXR or CT are the two main pillars for diagnosing pneumonia [23]. In patients with neutropenia, however, symptoms of pneumonia may not be apparent due to their compromised host immune responses [8, 11]; thus, diagnosis of pneumonia through imaging can be particularly critical [11, 12]. However, CXRs are reported to have limited diagnostic performance for pneumonia in FN patients [14, 15]. Furthermore, interpretation of CXR by an expert radiologist may not be readily available for timely management. In this regard, CAD may help in the accurate and timely diagnosis of pneumonia in these patients. Indeed, CAD used in our study could identify pneumonia on CXRs with higher sensitivity than radiologists (higher sensitivity than four of five radiologists at the pre-defined threshold). High sensitivity of the CAD in a consecutively-collected CXRs reflecting the prevalence of pneumonia and the spectrum of CXR findings of the actual clinical situation suggests this CAD can accurately identify pneumonia among CXRs of febrile neutropenia patients in the situation of daily practice.

Contrarily, CAD showed lower specificity than radiologist at the predefined threshold. The results suggest that the standalone interpretation by CAD without modification of the threshold may produce more false-positive results than radiologists’ interpretation. However, sensitivity may have priority over specificity for diagnosing pneumonia in FN patients, considering that pneumonia is frequently overlooked on CXR [11, 12, 26] and is associated with poor prognosis in FN [2, 3]. At the same sensitivity level as that of radiologists’ interpretation, CAD exhibited higher specificity than most radiologists (four of five). The result suggests that adjustment of the threshold for positive results can improve specificity while maintaining radiologist-level sensitivity or higher.

Calibration of a deep learning-based algorithm is an important factor for its interpretability and credibility, but this aspect is frequently overlooked [27]. In our study, we found that CAD tended to overestimate the probability of pneumonia (Fig. 2B). The same CAD system exhibited a similar tendency of overestimation for the identification of referable abnormalities in CXRs obtained from patients in the emergency department [28]. Recalibration of the CAD result may correct the overestimation and improve the interpretability and credibility of CAD [28].

Assisting radiologists to enhance their performance is another key role of CAD [29]. In our study, average sensitivity of radiologists was significantly improved at similar specificity in the interpretation with CAD. The results suggest that CAD can help in improving the quality of radiologists’ interpretation and management of patients. Preserved specificity while improving sensitivity suggests that false-positive detection by CAD can be appropriately arbitrated by radiologists. Improvement of inter-reader agreement in the interpretation with CAD suggests that the CAD can also reduce variability across radiologists in CXR interpretations. Prioritization of CXRs with high suspicion for critical finding is another potential application of CAD [30]. The relatively clear separation of CAD results between CXRs with and without pneumonia (Fig. 2C) suggests that such prioritization of CXR requiring early interpretation by a radiologist might be feasible with CAD. However, our retrospective study was not conducted for that purpose; further studies on this topic are warranted in the near future.

In patients with available chest CT as a firm reference standard for pneumonic infiltrates, CAD exhibited similar performance as that reported in a previous study, where the same CAD system was evaluated in patients suspected of having coronavirus disease (sensitivity 83.8% and specificity 59.3% in our study; sensitivity 81.5% and specificity 52.3% in the previous study) [31], suggesting the robustness of performance regardless of population characteristics. CAD exhibited slightly lower performances in patients who underwent chest CT than in patients who did not undergo chest CT although significant difference was not shown. This result is probably because chest CTs were obtained in patients with a questionable diagnosis of pneumonia or other causes of fever [1, 11], while patients with a definite diagnosis of pneumonia on CXRs or those with clearly normal CXRs and no clinical suspicion of pneumonia may not have undergone CT.

Our study also showed that CAD missed a substantial proportion of pulmonary infiltrates (16.2%) on CXRs, which could be identified on CT. Although sensitivity improvement occurred in some radiologists, they remained below 90%. Therefore, the negative results by the CAD or radiologist may not necessarily indicate the absence of pneumonia, and CAD cannot be a substitute for chest CT. Chest CT should be performed in patients with persistent fever despite empirical antibiotics, or those with clinical suspicions of pneumonia but negative CXRs [1, 11, 32, 33].

Consistency of performance in patients with different characteristics is an important factor for the reliability of CAD [29]. In our study, CAD exhibited similar performances between men and women. However, it exhibited lower specificities in older patients (age ≥ 60 years) than in younger patients, and CXRs obtained using a portable scanner than those obtained using a fixed radiography unit. Suboptimal image qualities of portable radiography and patient-related artifacts (e.g., limited inspiration and inappropriate positioning of patients) might have led to lower specificities. Previous studies reported that the identification of pulmonary infiltrates and differentiation from pleural effusion or atelectasis was difficult for human readers [34, 35]. A higher prevalence of existing pulmonary abnormalities (e.g., scarring, interstitial fibrosis, and emphysema) may also have contributed to more false-positive detection by CAD. A higher rate of false-positive detection should be considered when using CAD in clinical practice.

Our study has several limitations. First, because CAD was validated on a retrospectively collected dataset, the effect of CAD on the management and outcome of patients could not be evaluated. Second, since the reader test was conducted outside the daily practice, the reading behaviors of radiologists may be different from those in their daily practices. Third, since our study was performed in a single tertiary medical center, the generalizability of the results remains questionable. Finally, interpreting same CXRs twice in the reader test may have induced improvement of radiologists’ performance in the second reading session, regardless of using the CAD. Therefore, it cannot be excluded that the added value of the CAD has been overestimated.

## Conclusions

In conclusion, the deep learning-based CAD system exhibited radiologist-level performance in the identification of CXRs with pneumonia in consecutive FN patients. It enhanced the performance of radiologists for the identification of pneumonia. We believe that CAD system helps diagnose and manage pneumonia in FN patients.

## Supplementary Information

**Additional file 1**.** Supplementary information**. Development and function of the computer-aided detection sytem.** Figure S1**. Example input and output of the computer-aided detection system.** Table S1**. Performance of the computer-aided detection system at different thresholds.** Table S2**. Performances of the computer-aided detection system and radiologists in the reader test.

**Additional file 2**. Raw data analyzed in the study.

## Data Availability

All data generated or analyzed during this study are included in this published article and its supplementary material, except for chest X-ray images. Chest X-ray image data analyzed during the current study are not publicly available due to private health information policy of the institution but are available from the corresponding author on reasonable request.

## Abbreviations

ANC: Absolute neutrophil count
AUC: Area under receiver operating characteristic curve
CAD: Computer-aided detection
CI: Confidence interval
CT: Computed tomography
CXR: Chest X-ray
FN: Febrile neutropenia
IQR: Interquartile range
