# Supplementary material for: Deep learning computer-aided detection system for pneumonia in febrile neutropenia patients: a diagnostic cohort study
Source: BMC Pulm Med. 2021 Dec 7;21:406. doi: 10.1186/s12890-021-01768-0 (PMC8650735; doi:10.1186/s12890-021-01768-0)
Supplement: Supplementary file 2 — Additional file 2. Raw data analyzed in the study. [file 12890_2021_1768_MOESM2_ESM.docx]

**Supplementary Material**

**Development and function of the computer-aided detection (CAD) system**

The computer-aided detection (CAD) system tested in the present study (Lunit INSIGHT CXR 2, version 2.0.0.0, Lunit Inc., Seoul, Korea) was developed using deep convolutional neural network. Although the original CAD system was trained to identify findings of four major thoracic diseases (lung cancer, active pulmonary tuberculosis, pneumonia, and pneumothorax) on a chest X-ray (CXR), the commercialized CAD system was modified to detect three major thoracic abnormalities including pulmonary nodule, pulmonary infiltrate, and pneumothorax. The CAD was trained with 54,221 normal CXRs and 13,926 CXRs with lung cancer, 6,768 CXRs with active pulmonary tuberculosis, 6,903 CXRs with pneumonia, and 8,016 CXRs with pneumothorax. For the training process, pixel-level annotations for the location of abnormality on a CXR as well as image-level labels for the presence of abnormality were used.

The CAD system comprised three parallel classifiers that can detect pulmonary nodule, pulmonary infiltrate, and pneumothorax, respectively in an input CXR. Each classifier provided probability scores between 0 and 100% for the presence of target abnormality in the CXR. Afterwards, the CAD system provided maximum vale among the three probability scores for the presence of any of the three target abnormalities, as a final output. In addition to the probability score, the CAD system provided a heat map overlaid on the input CXR if the final probability score was 15% or greater, for the localization of the detected abnormality.

In the present study, the probability score for the pulmonary infiltrate was used instead of the final probability score for any of target abnormality, because the task was the identification of pneumonia.

The CAD system was approved for clinical used by the Ministry of Food and Drug Safety of Korea and commercially available in South Korea. Anyone who would like to test the CAD system can upload their own DICOM files as input and check the output results from the CAD at a website provided by the manufacturer (http://insights.lunit.io/) for free.

**References**

1. Hwang EJ, Park S, Jin KN, et al. Development and Validation of a Deep Learning-Based Automated Detection Algorithm for Major Thoracic Diseases on Chest Radiographs. JAMA Netw Open 2019; 2(3): e191095.

**Figure S1. Example input and output of the computer-aided detection (CAD) system**

A chest X-ray (CXR) from a patient with pneumonia was provided as an input for the computer-aided detection (CAD) system. The CAD system provided probability scores of 9%, 82%, and 1% for the presence of nodule, infiltrate, and pneumothorax, respectively. For the final output, the CAD system provided a probability score of 82% (maximum value of three probability scores for each target abnormality) for the presence of any target abnormality. In addition, the CAD system also produced a heat map for localization of detected pulmonary infiltrate at the left lower lung field.


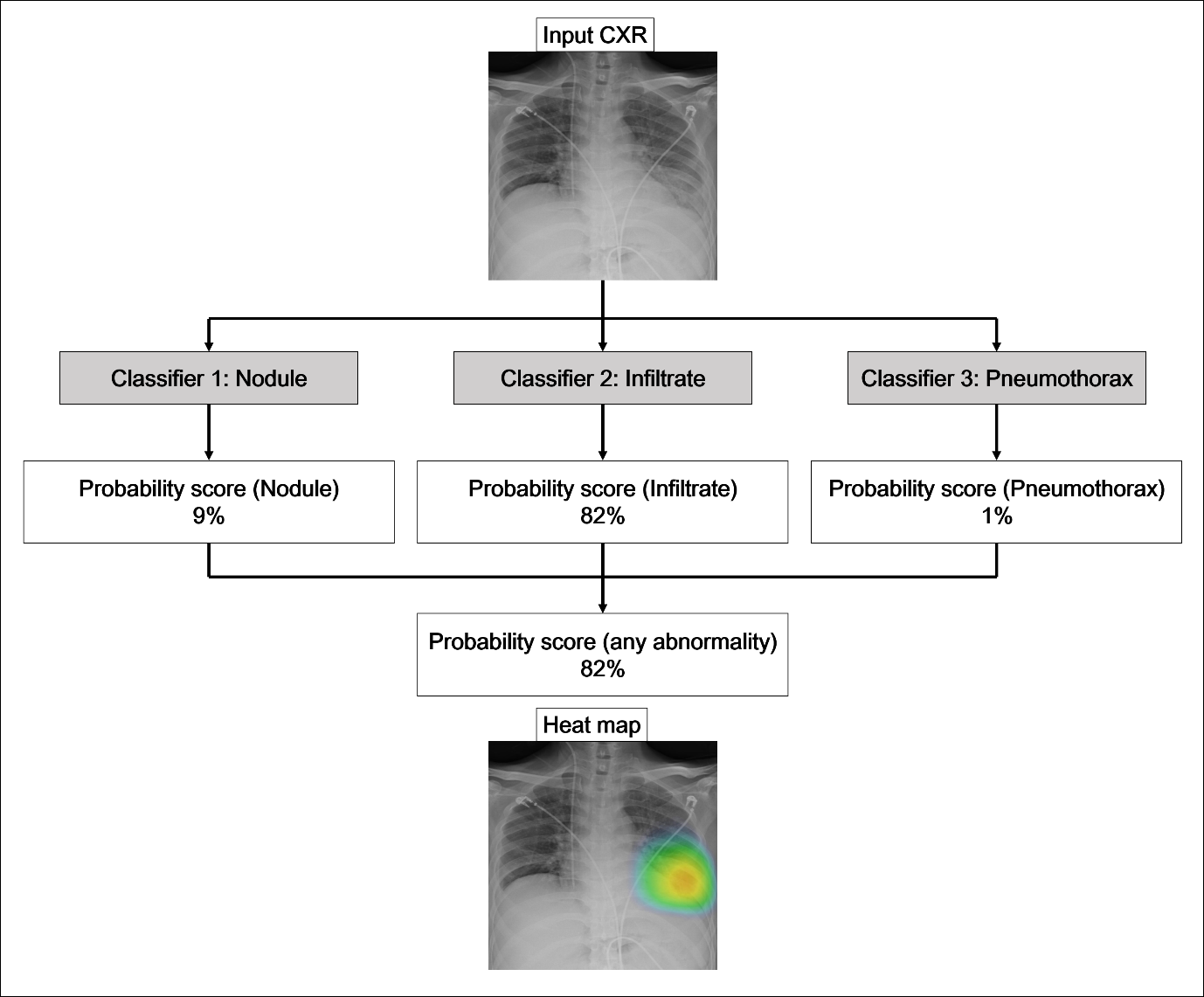


**Table S1. Performance of the computer-aided detection system at different thresholds**

| Threshold score | Sensitivity | Specificity |
| --- | --- | --- |
| 5% | 95.3% (122/128, 91.7–99.0%) | 49.6% (197/397, 44.7–54.5%) |
| 10% | 93.0% (119/128, 88.5–97.4%) | 60.5% (240/397, 55.6–65.3%) |
| 15% | 88.3% (113/128, 82.7–93.9%) | 68.3% (271/397, 63.7%–72.8%) |
| 20% | 87.5% (112/128, 81.8–93.2%) | 73.0% (290/397, 68.7–77.4%) |
| 25% | 85.9% (110/128, 79.9–92.0%) | 75.6% (300/397, 71.3–79.8%) |
| 30% | 85.2% (109/128, 79.0–91.3%) | 77.8% (309/397, 73.7–81.9%) |
| 35% | 82.8% (106/128, 76.3–89.3%) | 79.3% (315/397, 75.4–83.3%) |
| 40% | 81.3% (104/128, 74.5–88.0%) | 81.6% (324/397, 77.8–85.4%) |
| 45% | 80.5% (103/128, 73.6–87.3%) | 83.1% (330/397, 79.4–86.8%) |
| 50% | 79.7% (102/128, 72.7–86.7%) | 84.9% (337/397, 81.4–88.4%) |
| 55% | 77.3% (99/128, 70.1–84.6%) | 86.6% (344/397, 83.3–90.0%) |
| 60% | 75.0% (96/128, 64.5–82.5%) | 88.2% (350/397, 85.0–91.3%) |
| 65% | 72.7% (93/128, 64.9–80.4%) | 89.7% (356/397, 86.7–92.7%) |
| 70% | 71.1% (91/128, 63.2–78.9%) | 90.9% (361/397, 88.1–93.8%) |
| 75% | 68.0% (87/128, 59.9–76.1%) | 92.2% (366/397, 89.6–94.8%) |
| 80% | 64.1% (82/128, 55.8–72.4%) | 93.7% (372/397, 91.3–96.1%) |
| 85% | 55.5% (71/128, 46.9–64.1%) | 95.7% (380/397, 93.7–97.7%) |
| 90% | 49.2% (63/128, 40.6–57.9%) | 97.5% (387/397, 95.9–99.0%) |
| 95% | 34.4% (44/128, 26.1–42.6%) | 99.7% (396/397, 99.3–100%) |

Numbers in parentheses indicate numerators/denominators, 95% confidence intervals.

**Table S2. Performances of the computer-aided detection system and radiologists in the reader test**

| Reader | Sensitivity | *P*-value | Specificity | *P*-value |
| --- | --- | --- | --- | --- |
| CAD system | 86.6% (58/67, 76.2–92.9%)^a^ | Reference | 64.8% (127/196, 58.1–71.5%)^a^ | Reference |
| *Radiologist-alone interpretation* | | | | |
| Radiologists except Radiologist C (low sensitivity outlier) | 78.0% (209/268, 68.3–85.3%) | <.001^b^ | 72.2% (566/784, 67.5–76.5%) | .002^b^ |
| Radiologists except Radiologist C (high sensitivity, low specificity outlier) | 72.4% (53/67, 62.5–80.5%) | <.001^b^ | 83.0% (651/784, 78.4–86.8%) | <.001^b^ |
| *Interpretation with CAD* | | | | |
| Radiologists except Radiologist C (low sensitivity outlier) | 81.0% (217/268, 71.3–87.9%) | .039^c^ | 74.1 (581/784, 69.0–78.6%) | .081^c^ |
| Radiologists except Radiologist C (high sensitivity, low specificity outlier) | 77.6% (51/67, 67.8–85.1%) | .004^c^ | 82.7% (648/784, 77.8–86.6%) | .655^c^ |

Abbreviations: CAD, computer-aided detection

Numbers in parentheses indicate numerators/denominators, 95% confidence intervals.

^a^Performance of the CAD system at the predefined threshold (probability score of 15%)

^b^Comparison of performance between the CAD system and the radiologist-alone interpretation

^c^Comparison of performance between radiologist-alone interpretation and interpretation with CAD
